# Supplementary material for: Weakly coordinated Li ion in single-ion-conductor-based composite enabling low electrolyte content Li-metal batteries
Source: Nat Commun. 2023 Jul 8;14:4047. doi: 10.1038/s41467-023-39673-1 (PMC10329658; doi:10.1038/s41467-023-39673-1)
Supplement: Supplementary file 1 — Supplementary Information [file 41467_2023_39673_MOESM1_ESM.pdf]

Supplementary Information

of

Weakly coordinated Li ion in single-ion-conductor-based composite enabling low electrolyte content Li-metal batteries

Hyeokjin Kwon[1], Hyun-Ji Choi[1], Jung-kyu Jang[2], Jinhong Lee[1], Jinkwan Jung[1], Wonjun Lee[3], Youngil Roh[1], Jaewon Baek[1], Dong Jae Shin[1], Ju-Hyuk Lee[1], Nam-Soon Choi[1], Ying Shirley Meng[4,5]*, Hee-Tak Kim[1,6]**

[1]Department of Chemical and Biomolecular Engineering, Korea Advanced Institute of Science and Technology (KAIST), Daejeon, Republic of Korea

[2]Energy Materials Research Center, Korea Research Institute of Chemical Technology (KRICT), Daejeon, Republic of Korea

[3]Department of Energy Engineering, School of Energy and Chemical Engineering, Ulsan National Institute of Science and Technology (UNIST), Ulsan, Republic of Korea

[4]Department of NanoEngineering, University of California at San Diego, San Diego, 92093, California, USA

[5]Pritzker School of Molecular Engineering, University of Chicago, Chicago, IL, USA

[6]Advanced Battery Center, KAIST Institute for the NanoCentury, Korea Advanced Institute of Science and Technology (KAIST), Daejeon, Republic of Korea

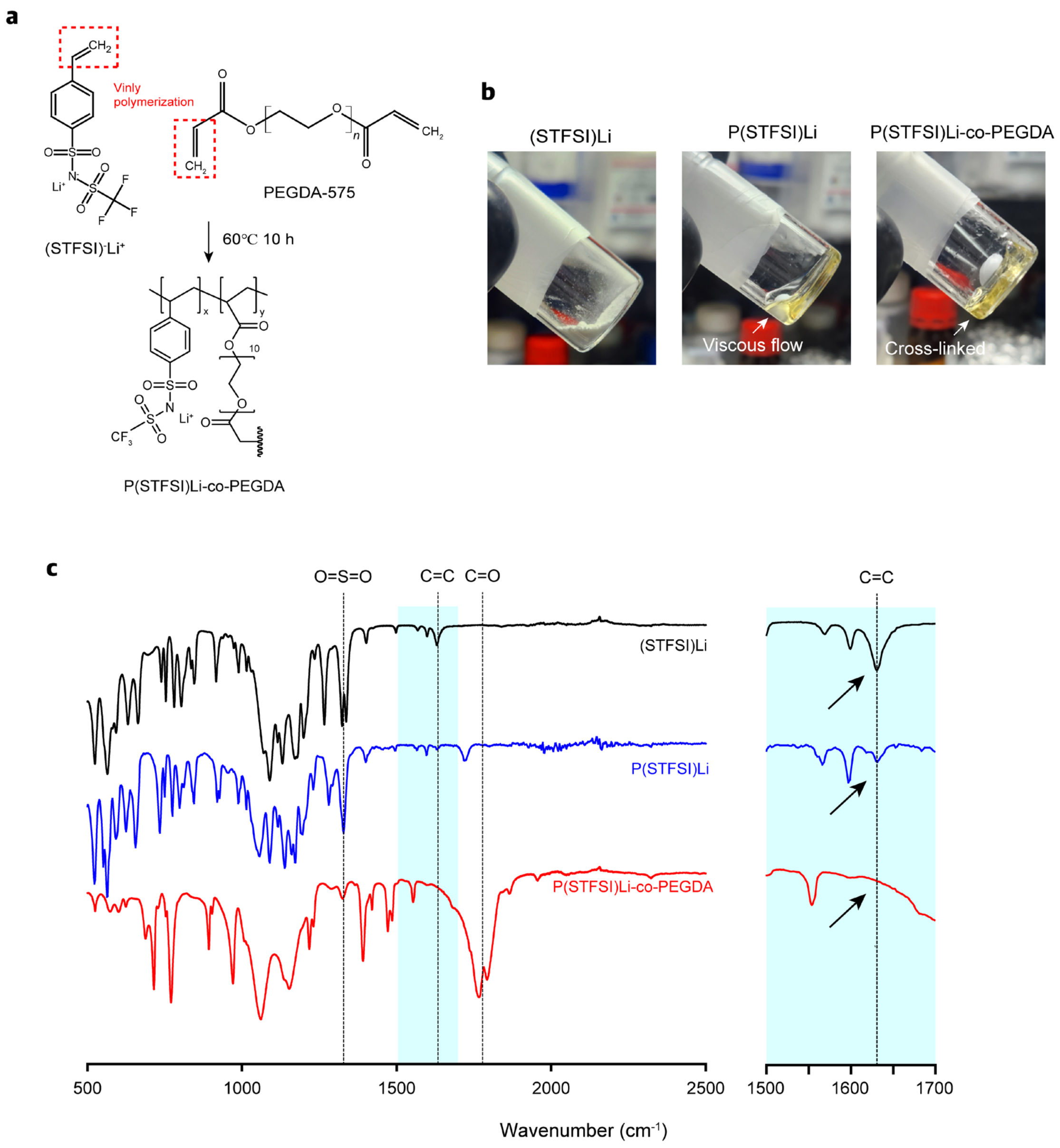

Supplementary Figure 1. Synthesis of S-PE (P(STFSI)Li-co-PEGDA). a, Chemical structures of (STFSI)Li, PEGDA and P(STFSI)Li-co-PEGDA. **b, c,** Optic images and FTIR spectra of (STFSI)LI, P(STFSI)Li, and P(STFSI)Li-co-PEGDA.

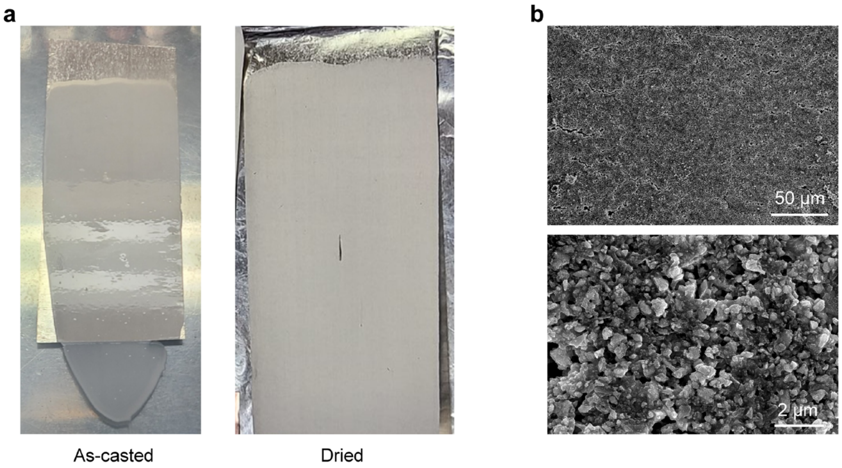

Supplementary Figure 2. Photographs and SEM of Li|S-CE/S-PE. a, Optic images of as-casted S-CE/S-PE on Li and dried S-CE/S-PE layer on Li. **b,** SEM images of S-CE/S-PE composite.

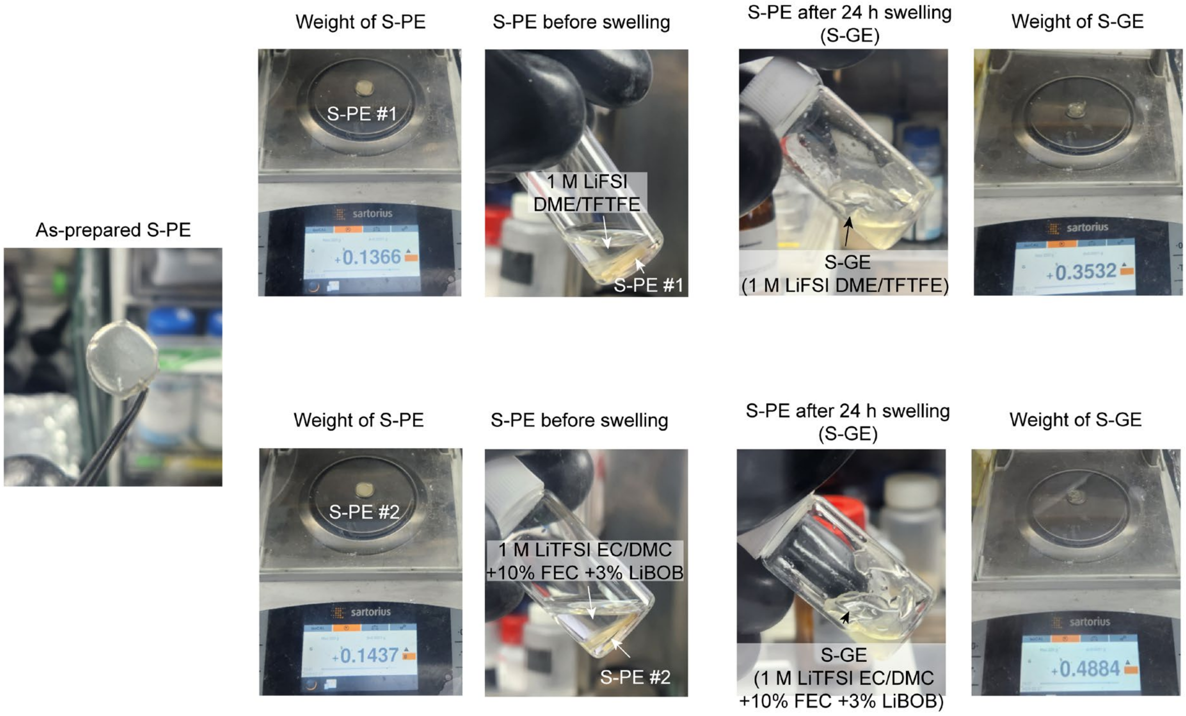

Supplementary Figure 3. Measurement of the equilibrium swelling ratio for S-PE.

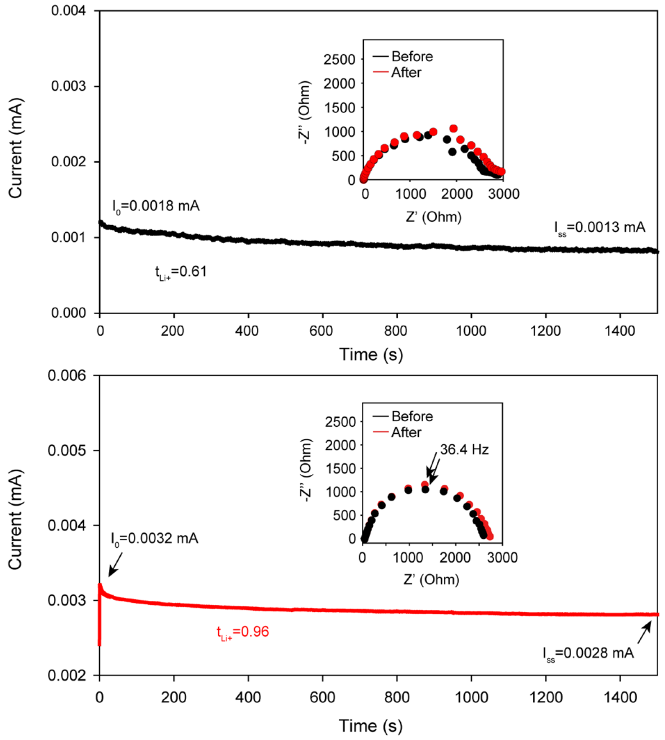

Supplementary Figure 4. Li$^+$ transference number. Li$^+$ transference number measurement for S-CE/B-PE (top) and S-CE/G-PE (bottom).

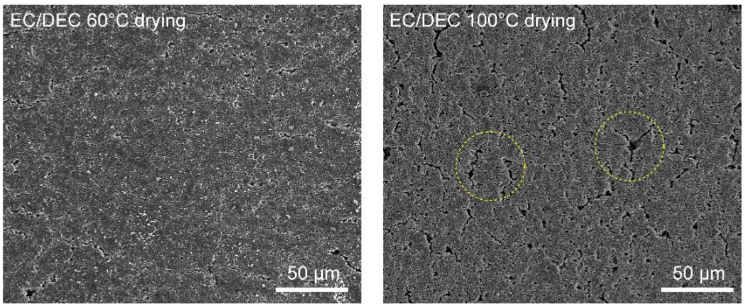

Supplementary Figure 5. Morphology of S-CE/S-PE composite layer at different drying temperatures.

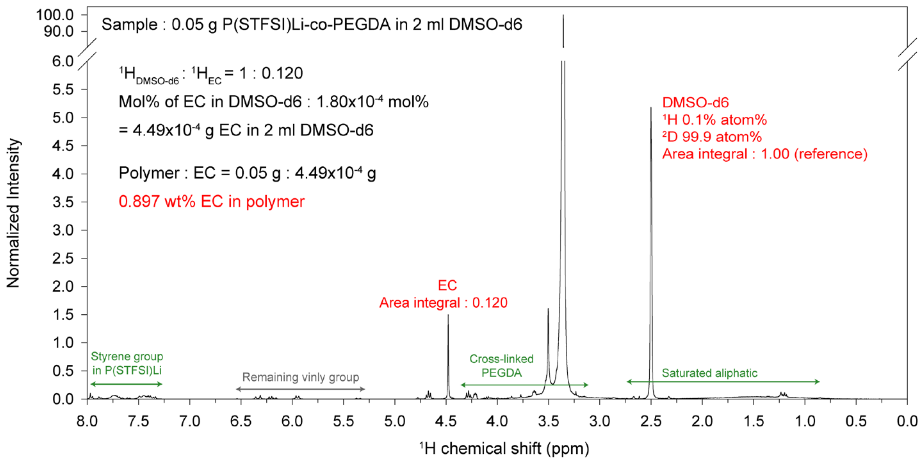

Supplementary Figure 6. NMR spectra of P(STFSI)Li-co-PEGDA in DMSO-d6. The amount of EC remaining after the drying process was detected through NMR spectra of a solution in which a polymer was dissolved in DMSO-d6.

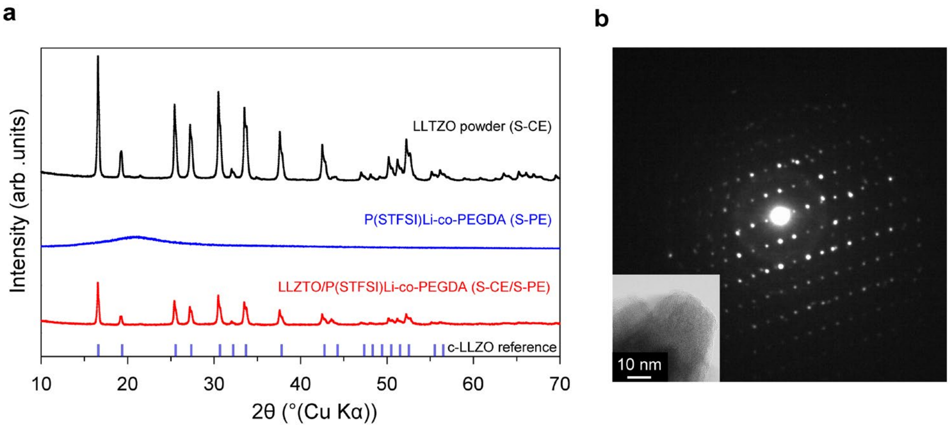

Supplementary Figure 7. XRD analysis for the S-CE/S-GE. a, X-ray diffraction spectroscopy (XRD) spectra of the pristine LLZTO powder (S-CE, 500 nm) and the fabricated S-CE/S-PE on SUS. **b,** Selected area electron diffraction (SAED) of S-CE/S-PE.

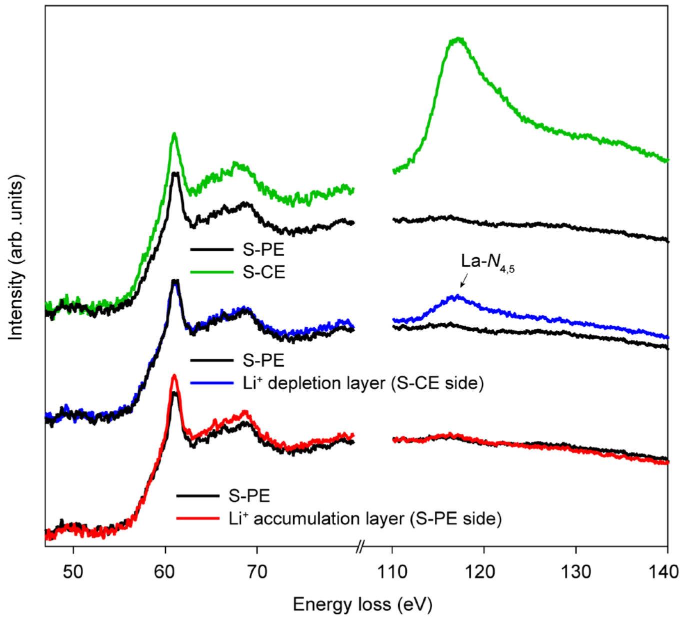

Supplementary Figure 8. EELS line spectra of S-CE/S-PE interface.

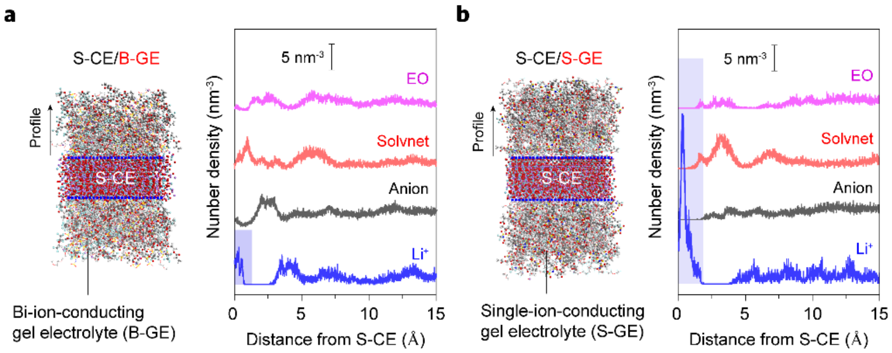

Supplementary Figure 9. Number density profiles at the ceramic/polymer interface. **a**, **b**, Snapshots of MD simulations and corresponding number density profiles of Li^+ and electrolyte molecules at the interface of S-CE/B-GE (a) and S-CE/S-GE (b). The zero position corresponds to the outmost layer of the S-CE.

Supplementary Note 1. Number density at the ceramic-polymer interface

For the S-CE/B-GE, anions in the B-GE are dragged to the interface by the coulombic interaction with the Li^+ accumulation layer, constituting a high number density (4.1 nm^{-3}, higher than that of bulk B-GE). In contrast, at the S-CE/S-GE interface, the number density of anions is low (1.1 nm^{-3}) because the anions in the S-GE are immobilized. The interface structure of the S-CE/B-GE resembles a diffuse double layer structure due to the mobility freedom of Li^+ and anions in the B-GE; in contrast, at the S-CE/S-GE interface, excess Li^+ forms a

compact Li^+ accumulation layer, because the anions cannot be redistributed in the S-GE phase to stabilize the Li^+ space charge. It appears that a large amount of solvent is attracted toward the S-CE/S-GE because anions are not attracted toward the interface of S-CE/S-GE. However, solvent molecules that are attracted significantly will feel greater steric hindrance to coordinate Li^+ than bulk liquids, which can make coordination relatively difficult. We believe that the Raman results we observed have increased free-DME for this reason.

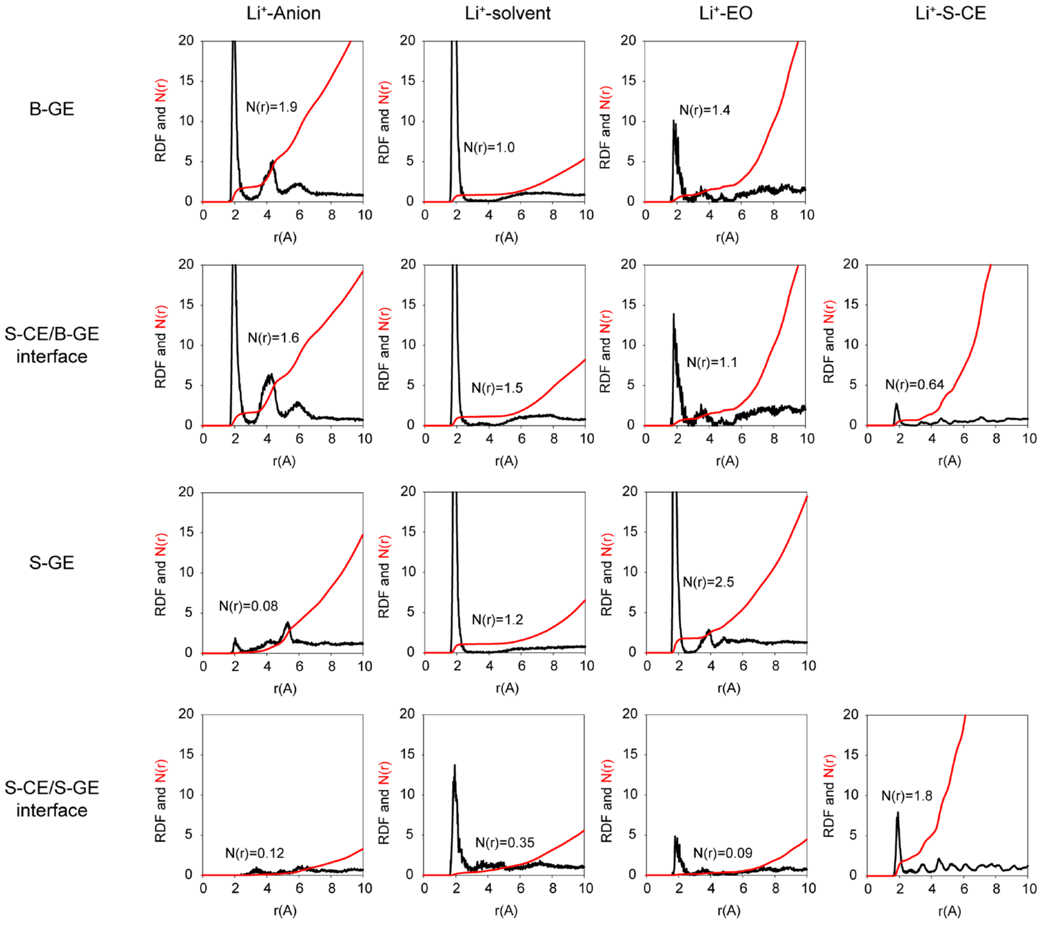

Supplementary Figure 10. Radial distribution function (RDF) and N(r) for the B-GE, S-CE/B-GE interface, S-GE, and S-CE/S-GE interface.

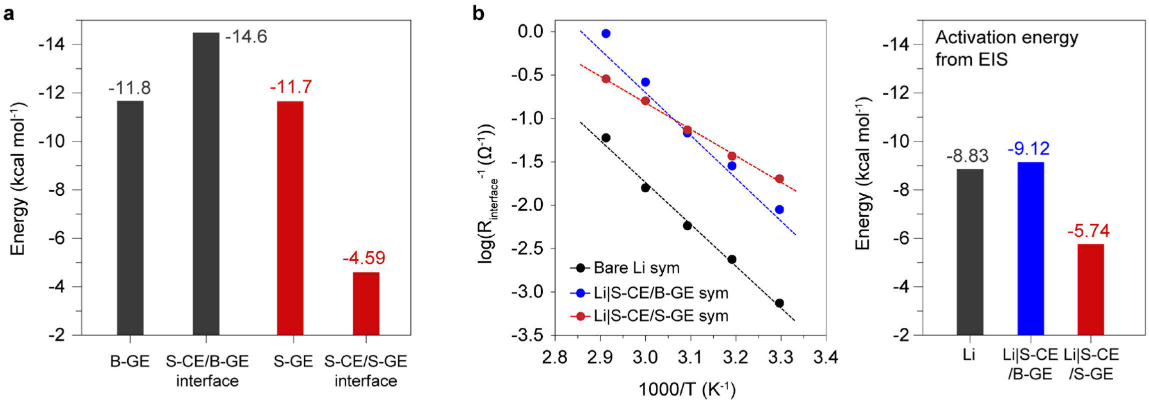

Supplementary Figure 11. Li$^+$ solvation free energy. a, Comparison of Li$^+$ solvation free energy calculated from MD simulation among B-GE, S-CE/B-GE interface, S-GE, and S-CE/S-GE interface. **b,** Arrhenius plot for the reciprocal of interfacial resistance for the Li and Li|S-CE/S-GE symmetric cell (left). Comparison of the activation energy determined from the interfacial resistances measured for the Li and Li|S-CE/S-GE symmetric cells (right).

Supplementary Note 2. Solvation free energy

To experimentally corroborate the weak coordination of Li$^+$, the Li$^+$ solvation free energy was calculated by a MD simulation for B-GE, S-CE/B-GE interface, S-GE, and S-CE/S-GE interface and the Li$^+$ de-solvation energy was measured by using impedance spectroscopy for the Li and Li|S-CE/S-GE symmetric cells. The B-GE, S-CE/B-GE interface and S-GE, showed similar solvation free energies (-11.8, -14.6, and -11.5 kcal mol^{-1}, respectively). The solvation free energy of the S-CE/S-GE interface was remarkably smaller (-4.59 kcal mol^{-1}) than those of the B-GE, S-CE/B-GE interface and S-GE, indicating a weakly coordinated Li$^+$ structure at the S-CE/S-GE interface. The peculiar coordination structure is dictated by the Li$^+$ de-solvation energy obtained from the impedance analysis. The activation energy for the interfacial charge transfer reaction was 5.74 kcal mol^{-1} for the Li|S-CE/S-GE electrode, which was lower than that S-CE/B-GE (9.12 kcal mol^{-1}) and without the S-CE/S-GE (8.83 kcal mol^{-1}). Since the de-solvation step is the rate-determining step for the Li electrode reaction, the activation energy corresponds with the de-solvation energy[1,2]. Therefore, the small de-solvation energy for the Li|S-CE/S-GE anode indicates that Li$^+$ in the S-CE/S-GE more loosely interacts with the surrounding solvent than B-GE.

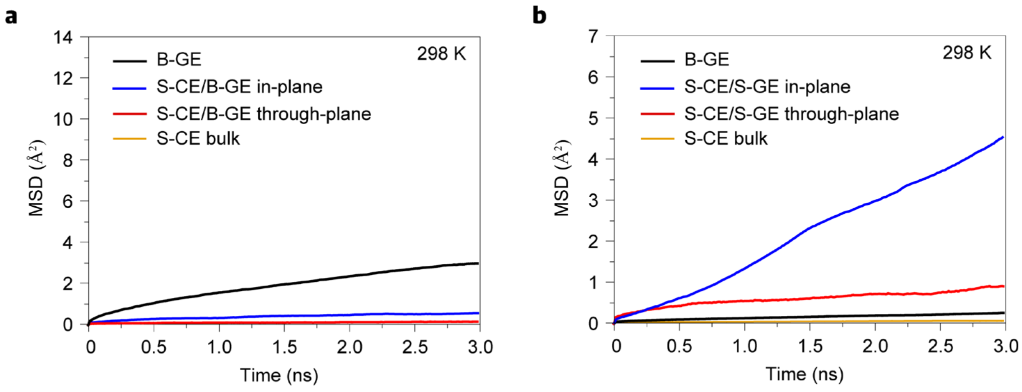

Supplementary Figure 12. Mean square displacement (MSD). MSD of Li^+ in S-CE/B-GE (a) and S-CE/S-GE (b) structures.

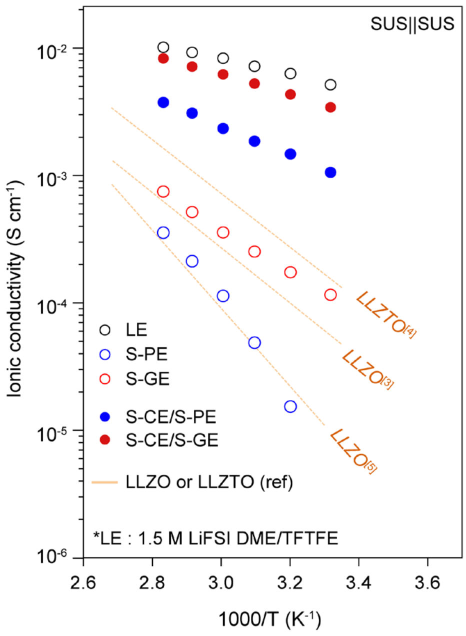

Supplementary Figure 13. Ionic conductivity and transference number. a, Ionic conductivities of liquid electrolyte (1.5 M LiFSI DME/TFTFE), S-PE, S-GE, S-CE/S-PE. S-CE/S-GE and literature values for ionic conductivity of LLZO and LLZTO[3-5]. **b,** Li^+ transference number measurement for S-CE/B-PE (top) and S-CE/G-PE (bottom).

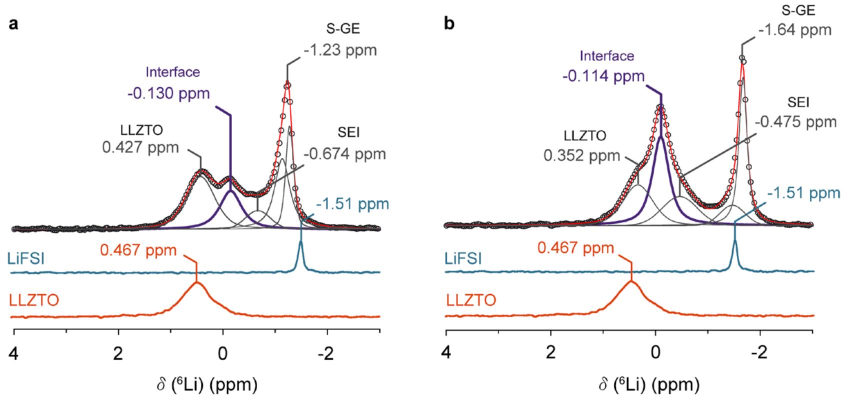

Supplementary Figure 14. ^{6}Li MAS-NMR spectra. a,b, ^{6}Li MAS-NMR spectra of S-CE/S-GE composite with liquid electrolyte before (a) and after (b) ^{6}Li exchange (0.05 mA cm^{-2} for 50 h).

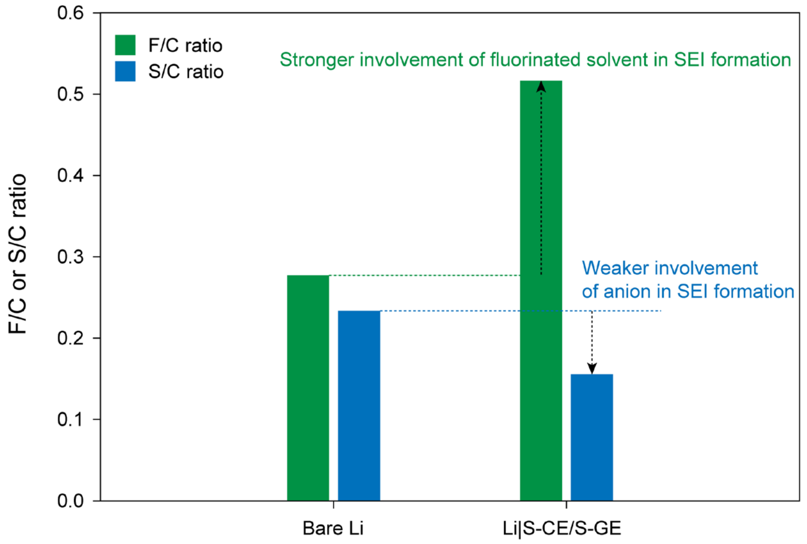

Supplementary Figure 15. Atomic F/C and S/C ratios of the SEI layers for bare Li and Li|S-CE/S-GE in 1.5 M LiFSI DME +50% TFTFE.

In the SEI layer of Li|S-CE/S-GE, a high F/C ratio indicates a decrease in DME decomposition (weak organic by-products), and a decrease in the S/C ratio indicates a decrease in anion (FSI^-) decomposition.

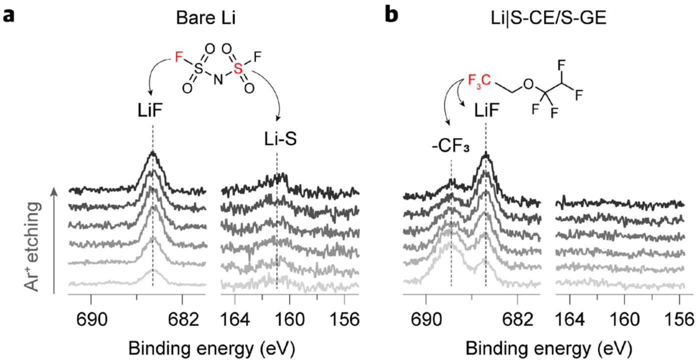

Supplementary Figure 16. F1*s* and S2*p* spectra of SEI layer in 1.5 M LiFSI DME +50% TFTFE. a, Bare Li. **b,** Li|S-CE/S-GE.

In the SEI layer of Li|S-CE/S-GE, no decomposition signal of anion was observed, whereas decomposition signal of fluorinated solvent was observed. This means that the fluorinated solvent, not the anion, contributes dominantly to the SEI.

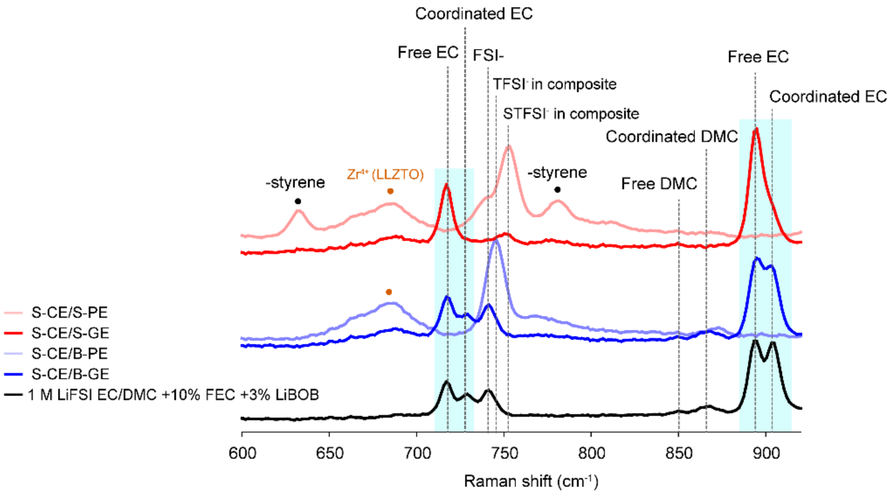

Supplementary Figure 17. Raman spectra before and after carbonate electrolyte permeation into the S-CE/B-PE and S-CE/S-PE. To distinguish anions in the liquid electrolyte and composite layer, LiFSI-based liquid electrolyte was used in this experiment.

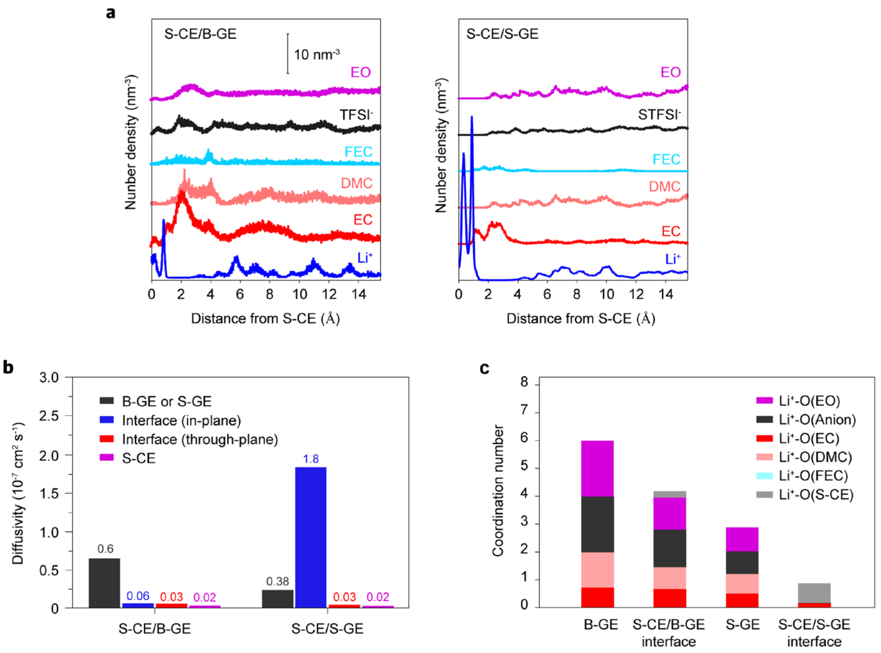

Supplementary Figure 18. MD simulations with carbonate electrolyte. a, b, Number density profiles of Li^+ and electrolyte molecules at the interface of S-CE/B-GE (left) and S-CE/S-GE (right). The zero position corresponds to the outmost layer of the S-CE. **b,** Diffusivities of Li^+ in S-CE/B-GE and S-CE/S-GE structures. The diffusivity of Li^+ at the interface was calculated isotropically in the in-plane and through-plane directions, respectively. **c,** Contributions to the coordination number of Li^+ in B-GE, S-CE/B-GE interface, S-GE, and S-CE/S-GE interface.

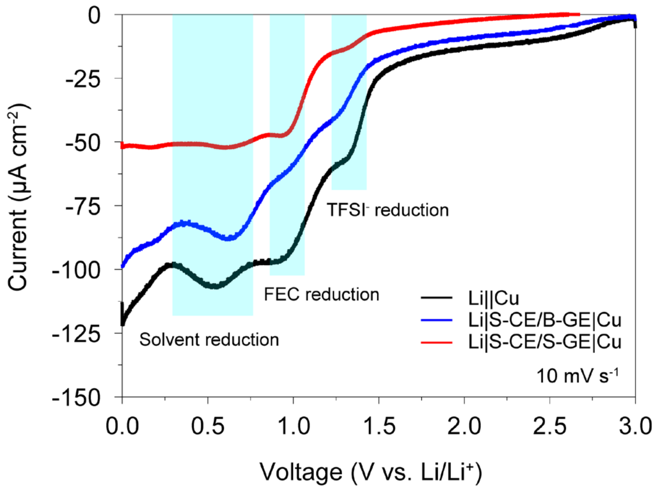

Supplementary Figure 19. Electrolyte reduction current via LSV in 1 M LiTFSI EC/DMC + 3% LiBOB 10% FEC.

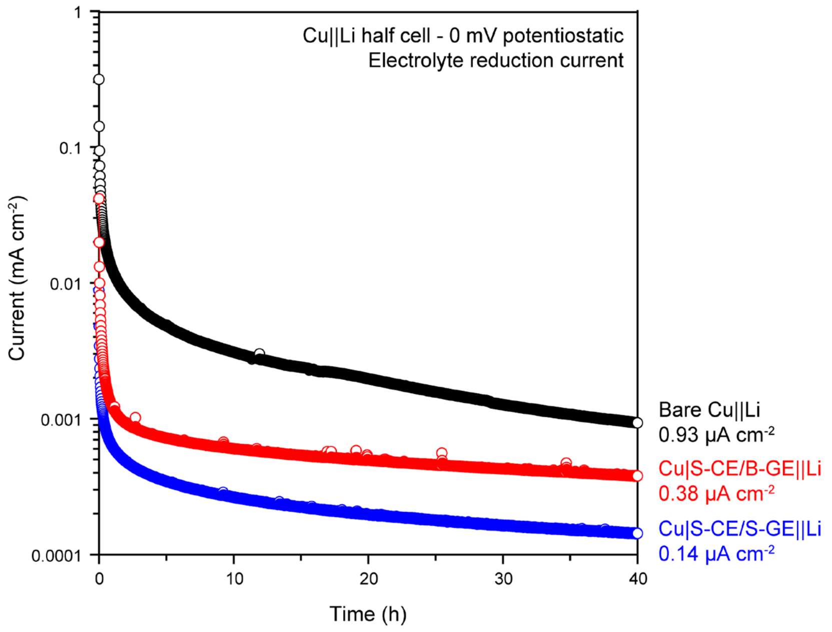

Supplementary Figure 20. Electrolyte reduction current via CA in 1 M LiTFSI EC/DMC + 3% LiBOB 10% FEC.

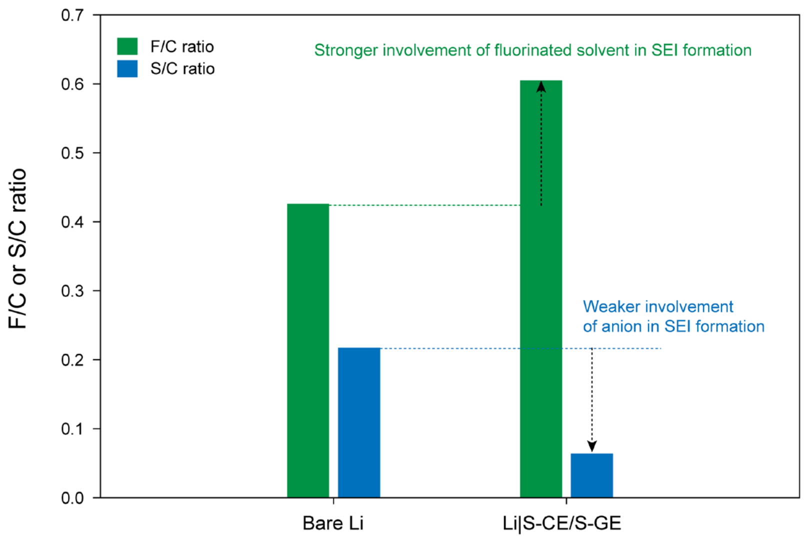

Supplementary Figure 21. Atomic F/C and S/C ratios of the SEI layers for bare Li and Li|S-CE/S-GE in 1 M LiTFSI EC/DMC +10% FEC +3% LiBOB.

In the SEI layer of Li|S-CE/S-GE, a high F/C ratio indicates a decrease in EC/DMC decomposition (weak organic by-products), and a decrease in the S/C ratio indicates a decrease in anion ($TFSI^-$) decomposition.

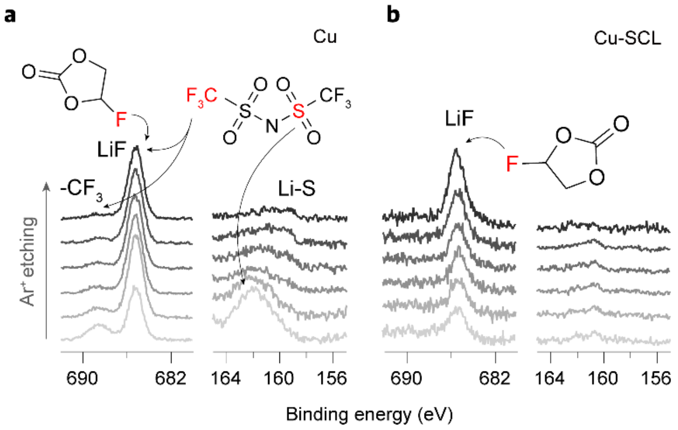

Supplementary Figure 22. F1*s* and S2*p* spectra of SEI layer in 1 M LiTFSI EC/DMC +10% FEC +3% LiBOB. **a,** Bare Li. **b,** Li|S-CE/S-GE.

In the SEI layer of Li|S-CE/S-GE, no decomposition signal of anion was observed, whereas decomposition signal of fluorinated solvent was observed. This means that the fluorinated solvent, not the anion, contributes dominantly to the SEI.

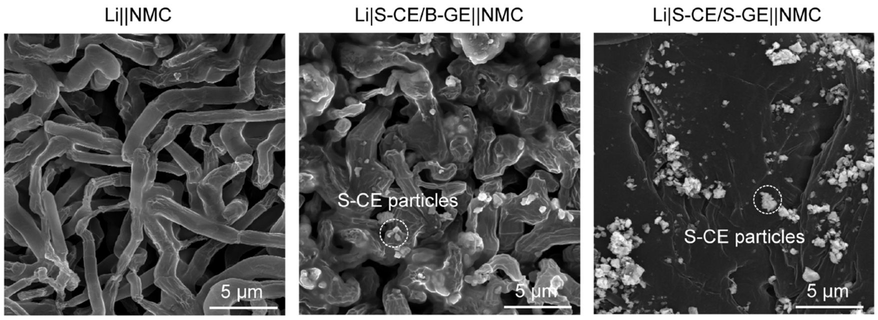

Supplementary Figure 23. Li morphology in 1 M LiTFSI EC/DMC + 3% LiBOB 10% FEC.

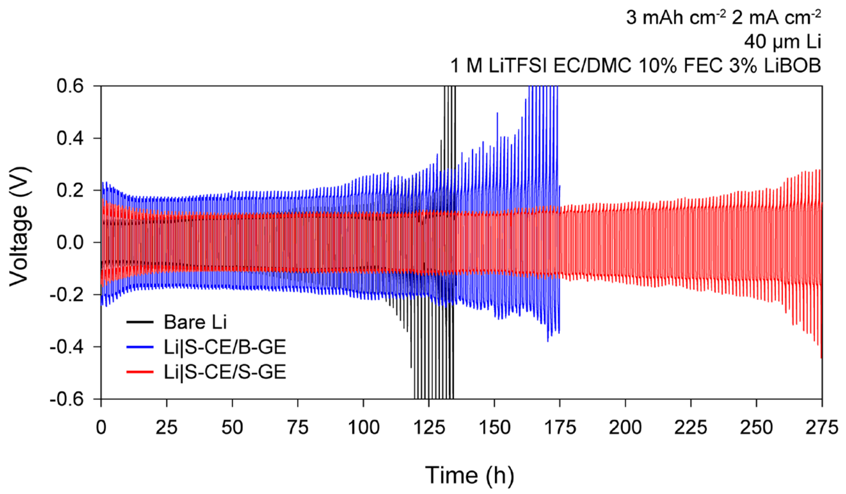

Supplementary Figure 24. Bare Li, Li|S-CE/B-GE, and Li|S-CE/S-GE symmetric cell at 2 mAh cm^{-2} and 3 mA cm^{-2} with carbonate electrolyte.

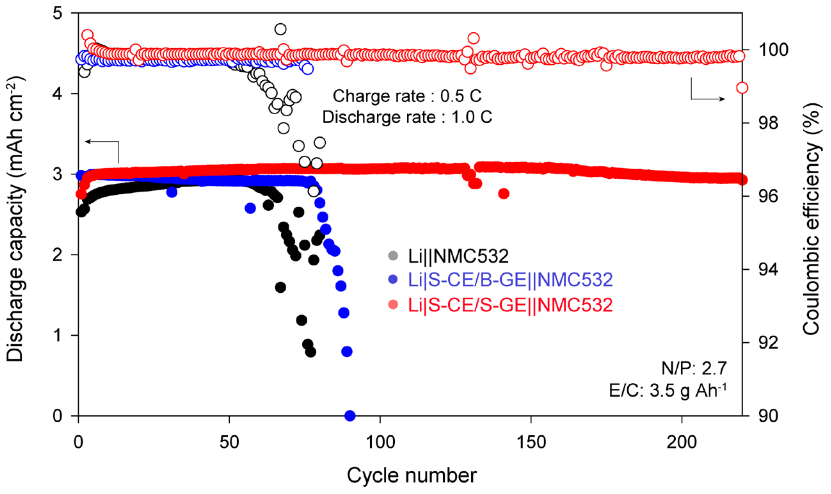

Supplementary Figure 25. Li||NMC532 full cell performance with 1.5 M LiFSI DME +50% TFTFE measured at 0.5 C constant current charging with 4.2 V cut-off voltage and 1.0 C discharging at 25 °C.

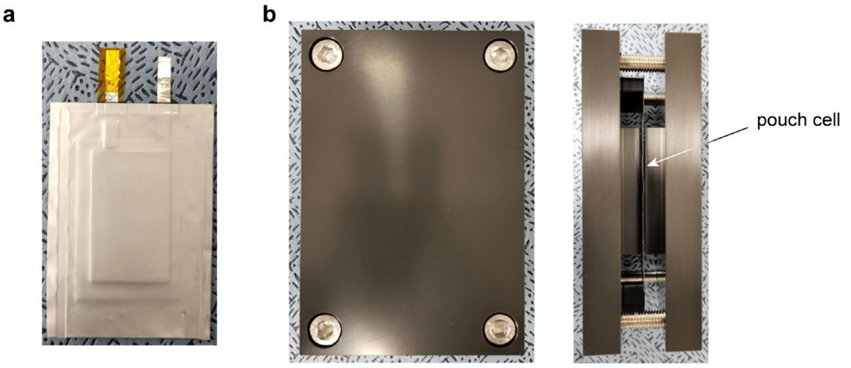

Supplementary Figure 26. Optic images of pouch cell with pressure jig. a, Prepared pouch cell. **b,** Top and side view of pressure jig.

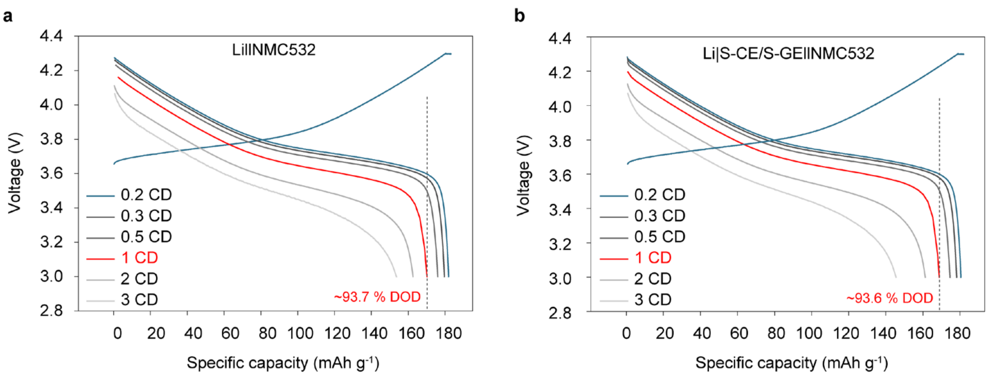

Supplementary Figure 27. Discharge rate capabilities of Li||NMC532 and Li|S-CE/S-GE||NMC532 full cells. a, Li||NMC532 **b,** Li|S-CE/S-GE||NMC532.

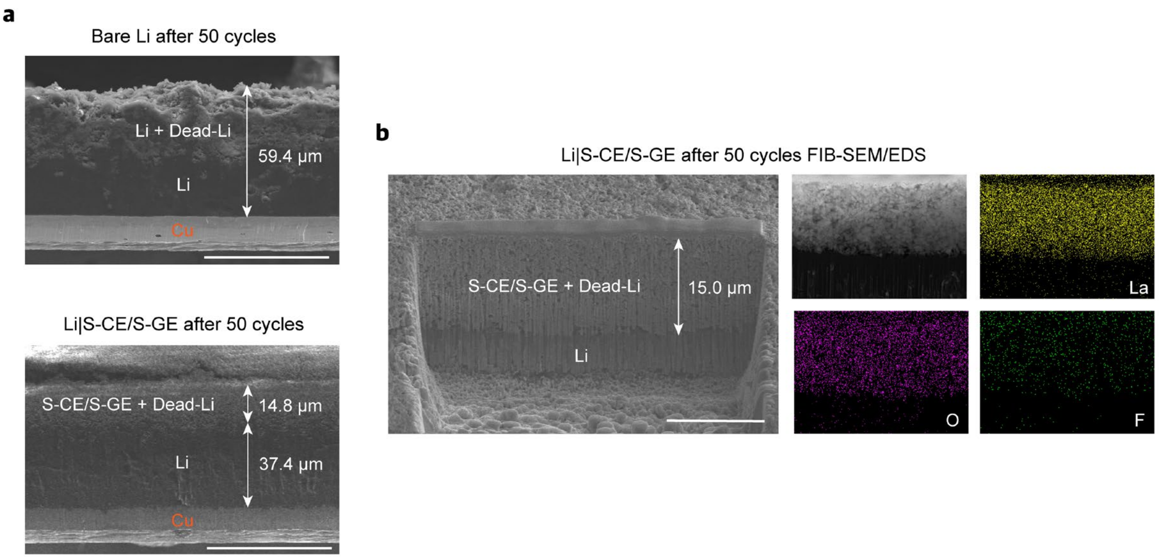

Supplementary Figure 28. Cross-sectional morphology of the cycled bare Li and Li|S-CE/S-GE. **a,** Cross-sectional SEM images. Scale bar: 50 μm. **b,** FIB-SEM (52° tilt) and EDS images for the cycled Li|S-CE/S-GE. Scale bar: 10 μm.

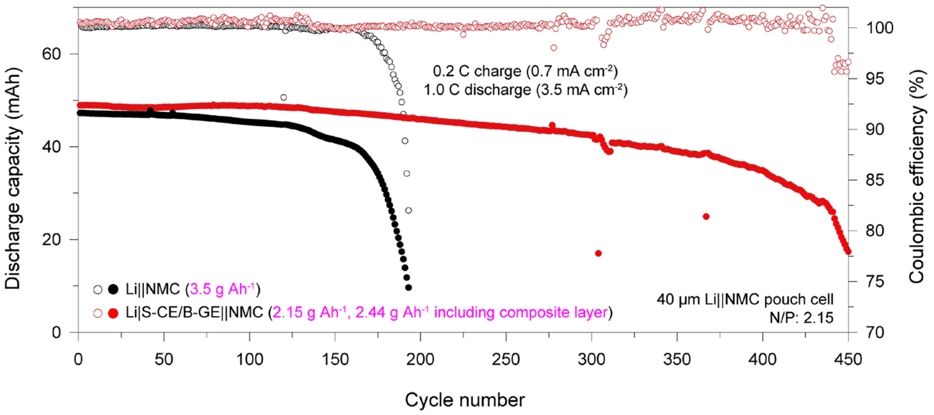

Supplementary Figure 29. Comparison of the cycling stability for the composite-layer-free Li||NMC cell with an E/C ratio of 3.5 g Ah^{-1} and the S-CE/S-GE (E/C ratio of 2.44 g Ah^{-1} including the mass of the composite layer).

Supplementary Table 1. Estimated gravimetric and volumetric energy densities of prototype pouch cells without and with S-CE/S-GE composite layer.

	Li‖NMC532	Li‖S-CE/S-GE ‖NMC532	Li‖S-CE/S-GE ‖NMC532
Al current collector (13 μm)	3.51 mg cm^{-2}	3.51 mg cm^{-2}	3.51 mg cm^{-2}
Cu current collector (8 μm)	7.17 mg cm^{-2}	7.17 mg cm^{-2}	7.17 mg cm^{-2}
Lithium metal (40 μm)	2.14 mg cm^{-2}	2.14 mg cm^{-2}	2.14 mg cm^{-2}
S-CE/S-GE layer (6 μm)	-	**1.07 mg cm^{-2}**	**1.07 mg cm^{-2}**
NMC532 cathode (61 μm)	21.47 mg cm^{-2}	21.47 mg cm^{-2}	21.47 mg cm^{-2}
PE separator (19 μm)	1.77 mg cm^{-2}	1.77 mg cm^{-2}	1.77 mg cm^{-2}
Electrolyte	E/C=2.15 g Ah^{-1}	E/C=2.15 g Ah^{-1}	E/C=1.28 g Ah^{-1}
Discharge capacity	3.72 mAh cm^{-2}	3.72 mAh cm^{-2}	3.72 mAh cm^{-2}
Average discharge voltage (0.1C)	3.82 V	3.82 V	3.82 V
Total stack energy (bi-cell)	28.41 mWh cm^{-2}	28.41 mWh cm^{-2}	28.41 mWh cm^{-2}
Total stack weight (bi-cell)	77.41 mg cm^{-2}	79.55 mg cm^{-2}	73.07 mg cm^{-2}
**Stack gravimetric energy density**	**367.0 Wh kg^{-1}**	**357.1 Wh kg^{-1}**	**388.8 Wh kg^{-1}**
**Stack volumetric energy density**	**1088.5 Wh L^{-1}**	**1040.7 Wh L^{-1}**	**1040.7 Wh L^{-1}**
**Cycle number**	**<50 cycles**	**400 cycles**	**100 cycles**

The gravimetric and volumetric energy density of the stacked bi-cells were calculated based on a previously suggested model[6]. The single bi-cell consists of one sheet of a double-side coated NMC cathode, one sheet of a double-side Li anode or double-side S-CE/S-GE composite-coated Li anode, two sheets of separator, and electrolyte.

References

1 Odziemkowski, M. & Irish, D. E. An Electrochemical Study of the Reactivity at the Lithium Electrolyte/Bare Lithium Metal Interface: I . Purified Electrolytes. *J. Electrochem. Soc.* **139**, 3063-3074 (1992).

2 Boyle, D. T. *et al.* Transient Voltammetry with Ultramicroelectrodes Reveals the Electron Transfer Kinetics of Lithium Metal Anodes. *ACS Energy Lett.* **5**, 701-709 (2020).

3 Wu, J. *et al.* Dense PVDF-type polymer-in-ceramic electrolytes for solid state lithium batteries. *RSC Adv.* **10**, 22417-22421 (2020).

4 Allen, J. L., Wolfenstine, J., Rangasamy, E. & Sakamoto, J. Effect of substitution (Ta, Al, Ga) on the conductivity of Li7La3Zr2O12. *J. Power Sources* **206**, 315-319 (2012).

5 Huang, M. *et al.* Effect of sintering temperature on structure and ionic conductivity of Li7−xLa3Zr2O12−0.5x (x=0.5~0.7) ceramics. *Solid State Ionics* **204-205**, 41-45 (2011).

6 Louli, A. J. *et al.* Diagnosing and correcting anode-free cell failure via electrolyte and morphological analysis. *Nat. Energy* **5**, 693-702 (2020).
